# The integration of training and off-training activities substantially alters training volume and load analysis in elite rowers

**DOI:** 10.1038/s41598-021-96569-0

**Published:** 2021-08-26

**Authors:** Gunnar Treff, Robert Leppich, Kay Winkert, Jürgen M. Steinacker, Benjamin Mayer, Billy Sperlich

**Affiliations:** 1 Division of Sports and Rehabilitation Medicine, University of Ulm, Leimgrubenweg 12, 89075 Ulm, Germany; 2 Software Engineering Group, Department of Computer Science, University of Würzburg, Würzburg, Germany; 3 Integrative and Experimental Exercise Science and Training, Institute of Sport Science, University of Würzburg, Würzburg, Germany; 4 Institute of Epidemiology and Medical Biometry, Ulm University, Ulm, Germany

**Keywords:** Physiology, Medical research

## Abstract

Training studies in elite athletes traditionally focus on the relationship between scheduled training (TRAIN) and performance. Here, we added activities outside of scheduled training i.e., off-training (OFF) contributing to total training (TOTAL) to evaluate the contribution of OFF on performance. Eight elite rowers recorded OFF and TRAIN during waking hours for one season (30–45 weeks) with multisensory smartwatches. Changes in performance were assessed via rowing ergometer testing and maximum oxygen uptake (${\dot{\text{V}}}$O_2max_). Based on 1-Hz-sampling of heart rate data during TRAIN and OFF (> 60% maximum heart rate (HR_max_), the volume, session count, intensity, training impulse (TRIMP), and training intensity distribution were calculated. OFF altered volume, TRIMP, and session count by 19 ± 13%, 13 ± 9%, and 41 ± 67% (p < 0.001). On an individual level, training intensity distribution changed in 3% of the valid weeks. Athletes exercised 31% of their weekly volume below 60% HR_max_. Low to moderate intensities dominated during OFF with 87% (95% CI [79, 95]); however, in some weeks high-intensity activities > 89% HR_max_ during OFF amounted to 21 min·week^−1^ (95% CI [4, 45]). No effect of OFF on changes of performance surrogates was found (0.072 > p > 0.604). The integration of OFF substantially altered volume, TRIMP, and session count. However, no effect on performance was found.

## Introduction

For decades, scientists and coaches have been seeking to identify the optimal dose–response relationship between various compositions of endurance training (i.e., exercise intensity, volume, frequency and its distribution over time) and performance outcome^1–6^. Interestingly, research in this area has almost exclusively highlighted the interaction of scheduled training routines and performance outcome but off-training activities are often overseen in elite athletes. Thus, such research lacks a holistic perspective on physical activity, which necessitates the integration of any activities during daily living and scheduled training with total training. The absence of a more complete perspective is astonishing because several off-training modulators of adaption (e.g. various recovery procedures, nutrition, activities of daily living including sedentariness) explain, at least to some degree, inter-individual adaptation among athletes^7^.

While there is a growing interest in the athletes' behavior outside of scheduled training, investigations tend to focus on health aspects and sedentary behavior^8–10^ rather than the combined ergogenic responses of training and off-training. In fact, healthy and physically active males (${\dot{\text{V}}}$O_2max_ 54 ± 7 mL•min^−1^•kg^−1^) can benefit from a high amount of habitual physical activity combined with prescribed endurance training^11^. However, little is known about the off-training activities of elite athletes such as rowers, who invest a substantial percentage (approximately 82%) of their waking time per year into off-training routines^6^ such as recovery procedures, activities of daily living (including sitting, lying, working, studying, active and passive transportation, and possibly additional exercise) and social engagements, with the remaining 18% invested into scheduled training. A recent pilot study of 11 national elite rowers concluded that rowers display considerable sedentary (< 1.5 metabolic equivalents (MET)) off-training behavior of more than 11.5 h•day^−1^, while at the same time engaging in 0.51 ± 0.44 h of activity of more than 6 MET on weekdays^9^. This is noteworthy since the 0.51 ± 0.44 h of such potentially “vigorous” activity and its possible ergogenic effect on the cardiovascular system is not integrated into the training analyses of scientists, coaches, or athletes.

To the best of our knowledge, off-training activity has never been considered in previous training studies as a potential contributor to biological adaption and/or training description in elite athletes. Consequently, the question arises of whether off-training activities alter descriptors of total training such as training intensity distribution, volume, and training load. Additionally, it remains unknown whether the inclusion of off-training with total training is a better descriptor for changes in performance over time. Therefore, the aims of the study were twofold: (i) to evaluate various descriptors of training and off-training characteristics in highly trained rowers over an entire season and (ii) to assess the contribution of off-training activity on performance outcomes through multiple training mesocycles.

## Methods

### Participants

The study originally comprised of 22 rowers (10 female), all competing at the national and international level. Due to the dropout of eleven participants and incomplete data for three athletes, a total of eight (two female) participants, with baseline characteristics summarized in Table 1, was included in the final analysis. Four athletes achieved the A-final of the U23-World Championships and three won medals. One athlete won a medal in the A-Final of the Elite World Championships, and one completed the C-Final (≥ rank 9). Two rowers ended their competitive careers after the study. The study was conducted according to the declaration of Helsinki and approved by the ethical board of the University of Ulm (##472/18). All participants gave informed written consent to participate in the study.

**Table 1:** Baseline characteristics of the eight rowers.

Variable	Male	Female
Number of participants	6	2
Age (years)	24 (20–29)	20–24
Body mass (kg)	82.6 (73.4–95.3)	54.5–62.8
Standing height (cm)	189 (181–194)	167–174
Power at 4 mmol/L blood lactate (W)	341 (291–381)	207–208
Maximum oxygen uptake (mL•min^−1^•kg^−1^)	73 (71–83)	56–66
2000 m ergometer test (m:ss.0)	6:12.1 (5:49.6–6:28.9)	07:25.2–07:29.1

Data are provided as median (minimum–maximum).

### Study design

**Figure 1:**
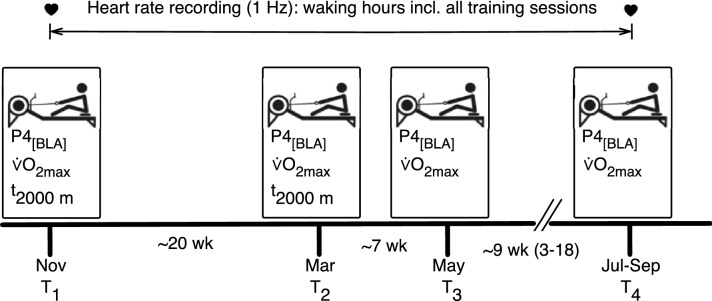
Study design. *P4*_*[BLA]*_ mechanical power output at 4 mmol/L blood lactate concentration; $\dot{V}$*O*_*2max*_ maximum oxygen consumption, *T*_*2000m*_ duration of 2000 m rowing ergometer test, *T*_*1*_*, T*_*2*_*, T*_*3*_*, T*_*4*_ timepoint of performance assessment.

The study commenced in November 2018 with the beginning of the preparation period and ended at each rower’s main competition, i.e., either the U23-world championships (n = 4; after 219 days), the senior championships (n = 2; 284 days), the European University Championships (n = 1; 318 days), or without a major international competition due to unsuccessful national qualification (n = 1; 213 days). Four ergometer tests were included throughout the observation period, comprising two 2000 m all-out tests (T_1_ and T_2_) as well as four assessments of maximum oxygen consumption (${\dot{\text{V}}}$O_2max_) and four incremental step tests (T_1_, T_2_, T_3_, T_4_; Fig. 1). T_1_ represents the beginning of the preparation period and T_2_ its end. T_3_ is was scheduled within the competitive season and T_4_ at its end. As a consequence, the time spans T_1_ to T_2_ and T_2_ to T_3_ were similar between participants (tests conducted within 140 ± 10 and 48 ± 5 days). The interval from T_3_ to T_4_ was adjusted individually in order to capture the end of the competitive season of each rower (59 ± 40 days). During the study period all athletes were instructed to wear a multisensory smartwatch (Polar M600, Kempele, Finland) to measure heart rate continuously during waking time to monitor all training and off-training periods.

### Testing

A series of tests was conducted on rowing ergometers (Concept II, Type D, Concept II, Morrisville, USA) to assess changes in participants’ physical performance and selected physiological variables.

#### Power output at 2 and 4 mmol·L^−1^ blood lactate

All rowers performed a 5 × 4-min incremental step test starting at 150 W in male light weight rowers, at 120 W in female, or 200 W in male open weight class rowers with 50 W (male) or 40 W (female) increments per stage. During a 30-s break between each stage, 20 µL of capillary blood were collected from the hyperemic earlobe and immediately analyzed amperometric-enzymatically (C-Line, EKF, Barleben, Germany). Specialized software was used to calculate mechanical power output at 2 and 4 mmol·L^−1^ [blood lactate] (P2_[BLa]_ and P4_[BLa]_) using a polynomic fitting of the power-lactate data (Winlactat, Mesics, Münster, Germany). P4_[BLa]_ is an accepted measure of rowing performance with standard errors of 1.4–3.3%^12^. We employed 3.3% as the lower limit to identify worthwhile changes, since the performance level of our rowers was similar to those in previous investigations^13^.

#### Assessment of maximum oxygen consumption

${\dot{\text{V}}}$O_2max_ was obtained at the same day of the incremental step test or one day later applying a ramp test protocol that enables a linear increase in power and objective test termination^14^. Briefly, target power of the rowing ergometer was initially set to 160 W and increased 30 W·min^−1^ (light weight male rowers and females) or 35 W·min^−1^ (male open weight class). The test automatically terminated when a rower failed to increase power within a 7 W threshold of five strokes. Oxygen consumption was measured with a metabolic analyzer employing dynamic mixing chamber technology (Metamax 3 ×, Cortex Biophysics, Leipzig, Germany). The technical error of measurement of this device is 0.11 L·min^−1^ (95% CI [0.03, 0.21])^15^. The system was calibrated prior to each test using ambient air and reference gas (16% O_2_, 5% CO_2_). A precision 3-L syringe (Hans Rudolph, Shawnee, USA) was employed to calibrate the flow sensor. ${\dot{\text{V}}}$O_2max_ was accepted when ${\dot{\text{V}}}$O_2_ failed to rise despite increasing work rate or an increase in ${\dot{\text{V}}}$O_2_ of ≤ 150 mL·min^−1^. Such a leveling-off or plateau ${\dot{\text{V}}}$O_2_ was found in all cases. In addition, respiratory exchange ratio at exertion in all rowers was > 1.1. ${\dot{\text{V}}}$O_2max_, defined as the highest ${\dot{\text{V}}}$O_2_ averaged over 30 s with increasing workload.

#### 2000-m ergometer test

To evaluate maximum rowing ergometer performance, all rowers performed an all-out 2000 m ergometer test 14 days after T_1_ and 19 days after T_2_, aiming to cover a virtual distance of 2000 m in the shortest possible time. Both 2000-m tests were part of the German federation’s standard testing procedure. Time was obtained from the rowing ergometer’s monitor (PM5, Concept II, Morrisville, USA) and converted to average power (P_2000m_). In elite rowing, this test is frequently employed to determine changes in maximum performance^12,16,17^. The error of P_2000m_ measurement with Concept 2 rowing ergometers is approximately 1.3% (95% CI [0.9, 2.9])^18^.

### Data assessment and management

All rowers wore a multisensory smartwatch allowing to measure heart rate (HR) at 1 Hz via optical heart rate measurement technology at the wrist and with a chest belt. Off-training heart rate was recorded with the optical wrist sensor. All training sessions were recorded with the chest belt, to avoid inaccurate measurements especially during rowing related to muscular contraction of the arm muscles. Participants were asked to label all periods of scheduled training in the smartwatch settings with the appropriate activity (e.g., rowing, cycling, running) and select the label *other indoor activity* for off-training heart rate recording. The setting *other indoor activity* allows sampling of heart rate at 1 Hz throughout off-training without activation of GPS, ensuring longer operational time. All athletes were instructed to upload their data at least once per week via the Polar Flow App (Polar, Oy, Finland) to the cloud-based Polar Flow software. Afterwards, all data were extracted as TCX files from each rower’s Polar Flow dashboards. The TCX files were parsed with the *ElementTree XML API* of the Python programming language (3.8) and stored in a MongoDB instance. Preprocessing and data analysis were conducted with the Python data analysis library Pandas (v.1.0.3).

### Labelling, intensity zones, and categorization of training activities

The labeling of the heart rate files (.fitfile) allowed grouping of training activities, abbreviated as TRAIN hereafter, including *rowing* (on water and ergometer), *non-specific endurance training* (running, spinning, cycling, cross-country skiing, etc.), *resistance training*, and *other* (stretching, yoga, etc.).

A 3-zone model following earlier categorization^5^ was applied with zone 1 ranging 60 < 82% of peak heart rate; zone 2, 82 < 88%; zone 3, 89 ≤ 100%. From here on, all training data with an intensity ≥ zone 1 will be abbreviated as TRAIN_≥z1_. Intensity zones were quantified according to the time-in-zone method^19^.

To calculate a combined measure of training volume and intensity, training impulse (TRIMP, a.U.) was calculated according to Bannister et al. as *duration x intensity*, where duration is given in minutes and intensity as zone 1–3^20^. The polarization index^21^ was calculated to categorize training intensity distributions and to evaluate possible changes over time.

### Off-training analysis

Daily waking activity other than training sessions was identified by the label *other indoor activity*, abbreviated hereafter as “OFF”. The smartwatch non-wear time was identified by the absence of any recorded data (i.e., time code and heart rate). A valid day was defined as a minimum of 480 min recorded per day^22^. We further strengthened this agreement by counting only those days with at least 480 min of daily activity recorded and any times marked as TRAIN. In cases in which one day was rated as *not valid,* all data were excluded from further analysis, as well as data from all weeks with less than six valid days. The sum of TRAIN and OFF is abbreviated as TOTAL hereafter.

To assess certain training descriptors (e.g., volume performed during OFF), only data with heart rate recordings ≥ zone 1 [i.e., ≥ 60% of peak heart rate (HR_max_)] were considered for further analysis. These data were also categorized into zones 1–3 according to the training intensity zones described above and will be denoted as OFF_≥z1_ hereafter. OFF_≥z1_ was used to calculate TRIMPS during off-training.

Since this is the first study to evaluate TRAIN and OFF, there is no previous agreement on how OFF sessions should be defined. Based on our own expertise, consultations with coaches and colleagues in the field, a session during OFF_≥z1_ was defined as any activity with a heart rate indicating an intensity ≥ zone 3 for a period of ≥ 1 min or an activity associated with an intensity ≥ zone 1 lasting ≥ 5 min. In case activities were separated by ≤ 5 min, any number of these activities were merged into one session. The rationale behind this categorization was not to disregard a 1-min intensity effort during OFF, because this duration corresponds to a typical interval length or for example, a high-intensity 400 m run. We also aimed to cumulate several high or low intensity intervals into one "session" and therefore arbitrarily defined the limit of 5 min.

### Statistical analysis

Mean values are given either as arithmetic mean ± standard deviation or median (minimum–maximum). After checking the normal distribution using the Shapiro–Wilk test, paired *t* tests assessed the difference between TOTAL vs. TRAIN for the training variables of intensity, volume, session, intensity distribution, polarization index, and TRIMPS. To evaluate changes in performance over time (i.e., changes in the four measurements T_1_–T_4_), a mixed model analysis was applied, including the time point of performance assessment as a fixed independent predictor and a random intercept accounting for the repeated measurements structure of the data. Pairwise differences between the single time points of measurement were assessed using linear contrasts in the mixed model.

To investigate whether the inclusion of off-training might explain changes in performance more accurately than training data only, we framed the training data between T_1_, T_2_, T_3_, and T_4_ in two different ways to calculate their effect on performance changes. First, we used *all* data of each training variable between T_1_, T_2_, T_3_, and T_4_. Since the period between the performance measurements varied among each athlete especially from T_3_ to T_4_ (due to participation in different championships or career ending), we applied a second framing procedure of the training data. Here we calculated the data of the preceding 21 days before T_2_, T_3_, and T_4_ to allow for a factual normalization of the same time span preceding each measurement for each athlete. Even though the interval of 21 days is arbitrary to some extent, we consider this range to be reasonable in order to reflect changes in performance better than accounting the complete period between measurements. Furthermore, this time span corresponds exactly to the minimum duration between two measurement times, namely for the athlete who terminated his career shortly after T_3_. The corresponding average values of both framings for TRAIN_≥z1_, OFF_≥z1_, and TOTAL_≥z1_ were then compared to the changes in performance (P2_[BLA],_ P4_[BLA],_ ${\dot{\text{V}}}$O_2max_) using a mixed linear model, allowing the identification of significant interactions between changes in performance and the different training measures. Specifically, each of the training variables mentioned above were included as a fixed independent predictor along with the time point of performance assessment accompanied by a random intercept. The mixed models were calculated with SAS (v. 9.5). *t* tests and all descriptive information were calculated using SPSS (IBM Corp. Released 2017. IBM SPSS Statistics for Macintosh, Version 25.0. Armonk, NY: IBM Corp). The threshold for significance was p ≤ 0.05 for all inferential analyses.

## Results

### Dataset

The entire dataset of eight rowers comprised 100,738,768 s, resulting in a total of 1,425 valid days and 194 valid weeks. This timeframe corresponded to 178.0 ± 45.8 days, or 24.3 ± 6.8 valid weeks per athlete, with an individual range (min–max) of 122–241 days and 16–34 weeks. On average, 88 ± 9% and 77 ± 11% of the possible days and weeks were recorded and rated as valid. The average observation period was 35.0 ± 6.0 weeks, ranging 30–45 weeks depending on the time point of the athlete’s main competition or dropout. Three athletes reported illness leading to training abstinence of 5, 8, and 22 days, respectively. Three other athletes reported injuries that hindered rowing training for 3, 6, and 14 days; rowing training was replaced by non-specific endurance training in these cases.

### Training data

Table 2 displays the average training volume and session count per week of all rowers. Rowing dominated (54%), followed by unspecific endurance and strength training. However, when analyzing the training volume for intensities ≥ zone 1, the average volumes of rowing and endurance training were 18% and 35% lower compared to all TRAIN data. Also, volume of TRAIN_≥z1_ was significantly lower (31%, p < 0.001) than TRAIN (Table 3). In other words, one third of all labeled TRAIN-activity was performed ≤ 60% HR_max_.

**Table 2:** Training volume and sessions spent in different training modes calculated for all logged training (TRAIN) data and exclusively for intensities equal or higher than 60% of maximum heart rate (TRAIN_≥z1_).

Domain	Variable	Volume		Sessions	
		Min/week	%	Sessions/week	%
TRAIN	Rowing	447 ± 227	54	6 ± 3	62
	Endurance	247 ± 295	30	3 ± 2	26
	Strength	109 ± 79	13	1 ± 1	10
	Other	26 ± 127	3	0 ± 1	2
TRAIN_≥z1_	Rowing	368 ± 197	67	6 ± 3	62
	Endurance	160 ± 184	29	3 ± 2	26
	Strength	23 ± 30	4	1 ± 1	10
	Other	2 ± 19	0	0 ± 1	2

Data are provided as arithmetic mean ± standard deviation or percentage within domain, respectively.

**Table 3:** Weekly averages of training descriptors in eight rowers during the study period (30–45 weeks).

Descriptor	p-value	Mean	sd	95% CI
**Entire recorded volume (min•week**^**-1**^**)**				
TOTAL	< 0.001	8052	1157	[7888, 8216]
TRAIN		806	372	[753, 859]
OFF		7246	1149	[7083, 7409]
**Training volume ≥ 60% HR**_**max**_** (min•week**^**-1**^**)**				
TOTAL_≥z1_	< 0.001	688	291	[647, 729]
TRAIN_≥z1_		554	254	[519, 591]
OFF_≥z1_		133	156	[111, 155]
**Session count (1•week**^**-1**^**)**				
TOTAL_≥z1_	< 0.001	17	9	[15, 18]
TRAIN_≥z1_		10	3	[9.5, 10.4]
OFF_≥z1_		7	8	[5, 8]
**TRIMPS (a.U.•week**^**-1**^^**-1**^				
TOTAL_≥z1_	< 0.001	646	307	[603, 690]
TRAIN_≥z1_		564	279	[525, 604]
OFF_≥z1_		82	118	[65, 99]

*TRAIN* training or activity data recorded during scheduled training, *OFF* recorded outside of TRAIN, *TOTAL* sum of TRAIN + OFF, the suffix _≥z1_ implies only data of intensities ≥ 60% of maximum heart rate (HR_max_), *TRIMP* training Impulse calculated as activity zone (1–3) times duration (minutes) adapted from Banister et al.^20^. p-values are based on paired *t* tests between TOTAL vs. TRAIN for each variable. Mean values are provided as arithmetic mean, *sd* standard deviation, *CI* confidence interval.

### Training (TRAIN) vs. total training (TOTAL)

The data in Table 3 indicate that TRAIN_≥z1_ comprised approximately 81% of TOTAL_≥z1_, or conversely, that OFF_≥z1_ contributed 19% of TOTAL_≥z1_. Furthermore, OFF_≥z1_ significantly and substantially contributed to 41% of the session count and to more than 11% of all TRIMPS. Finally, it is worth mentioning, that the addition of OFF_≥z1_ to TRAIN results in 806 + 133 = 939 min·week^−1^, indicating that OFF_≥z1_ contributes 14.2% to TRAIN.

### Training intensity distribution during training and off-training

**Figure 2:**
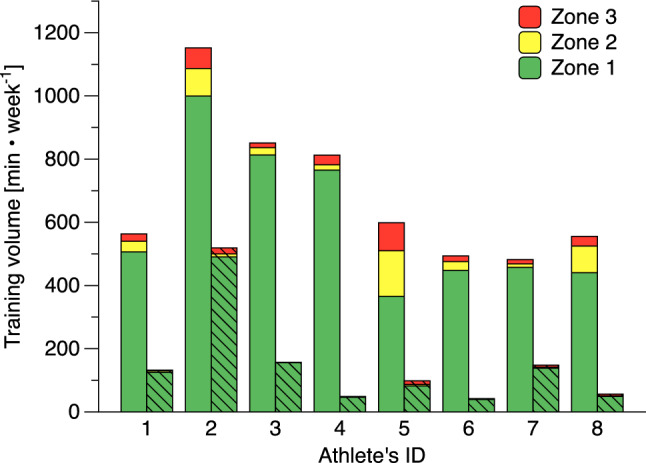
Training volume and intensity distribution during regular training (TRAIN, solid colors) and during all other waking hours (OFF, hatched) in eight national elite rowers. Zones refer to intensity zones, with zone 1 ranging from 60 ≤ 82% of maximum heart rate, zone 2 ranging 82 ≤ 88%, and zone 3 from 88 to 100% of maximum heart rate. The percentages of individual training intensity distributions (%Zone1–%Zone3–%Zone3) during TRAIN/OFF were #1: 90-6-4/95-4-2, #2: 87-8-6/95-2-4, #3: 96-3-2/99-0-0, #4: 94-2-4/95-2-3, #5: 61-24-15/84-5-12, #6: 91-6-4/94-5-1, #7: 95-2-3/94-2-4, and #8: 79-15-5/87-2-11.

The average individual volume during TRAIN was highest in athlete #2, with 1152 min·week^−1^; the remaining athletes ranged from 482 to 851 min·week^−1^ (Fig. 2). For rower #2, OFF-volume was considerably higher (519 min·week^−1^) than that of the other rowers (42–157 min·week^−1^). During TRAIN, percentages of zone 1 were in the range 87–96% for most rowers but considerably lower in two (79% and 61%). Two of the eight individual distributions (25%) were polarized (i.e., polarization index > 2.00 a.U.), although with a high percentage of low intensity training of at least 94%. During OFF, low intensity training was clearly dominant ranging from 84 to 99% in all rowers, with five (63%) OFF intensity distributions rated as polarized (Supplementary Table S1). The volume of high intensity training (i.e. zone 3, which is ≥ 88% of maximum heart rate) during OFF averaged 4.1 min·week^−1^ (range 0.5–19.1) (95% CI [0.5, 11.4]). The individual maxima of zone 3 training during OFF averaged 21 min·week^−1^ (6–81) (95% CI [4, 45]) in some weeks.

The percentages of zones 1, 2, and 3 were not significantly different between TRAIN_≥*z1*_ (84-8-7) and TOTAL_≥*z1*_ (84-8-8) (p-values: 0.776, 0.209, and 0.070). And even though the polarization index of the entire period differed significantly between TRAIN_≥*z1*_ and TOTAL_≥*z1*_ (1.75 ± 0.52 vs. 1.78 ± 0.49; p < 0.001), there was no meaningful change of the mean beyond the limit of 2.00 a.U. (i.e., the threshold differentiating polarized from non-polarized distributions). However, further analysis revealed a meaningful change in the polarization index (i.e., from ≤ 2.00 a.U. to > 2.00 a.U. or vice versa) occurred in 6 of 194 weeks for five rowers when considering TOTAL_≥z1_. In 5 of these 6 weeks (83%), a non-polarized distribution changed to a polarized, with the opposite occurring in 1 of 6 weeks (17%). These meaningful changes occurred before T_2_ and T_3_, as well as before T_4_.

**Figure 3:**
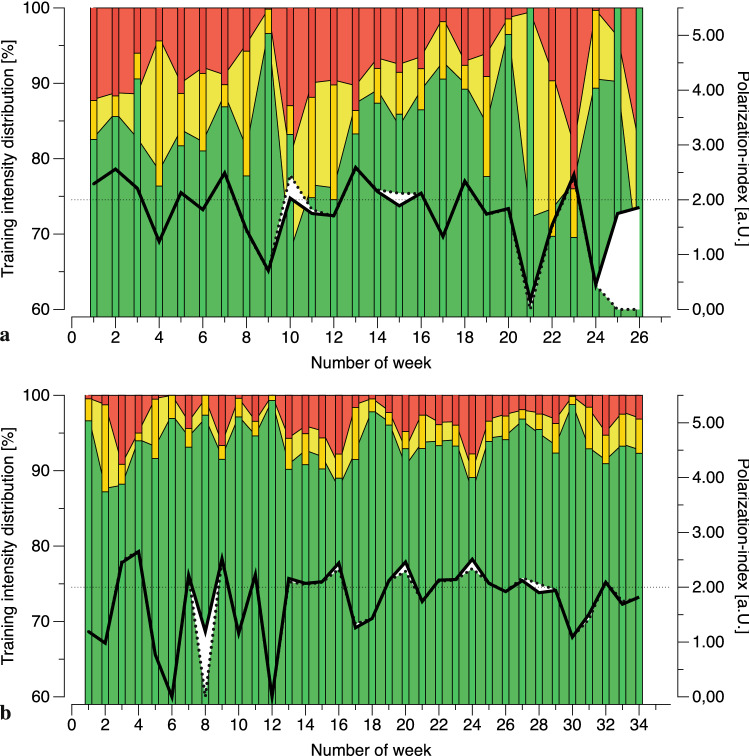
(**a**,**b**) Exemplary analysis of two athletes’ training monitoring over a period of 26 (**a**) and 34 (**b**) weeks. Green, yellow and red bars indicate the training intensity distributions (TID) as percentages of the labeled training (TRAIN_≥z1_) assigned to zones 1 (60 ≤ 82% of maximum heart rate, green), zone 2 (82 ≤ 88%, yellow), and zones 3 (88–100%, red), respectively. The background areas in similar colors indicate the TID, derived from the total training volume (i.e., labeled training plus activities during daily living with heart rates ≥ 60% of the maximum, TOTAL_≥z1_). The black line indicates the polarization index of the total training (according to Treff et al.^21^) while the dashed line shows the labeled training, only. The shaded area highlights differences in the polarization index between the labeled and the total training.

Figure 3a,b illustrates two examples of differences in intensity distributions between TRAIN_≥*z1*_ (bars) and TOTAL_≥*z1*_ (background). Figure 3a (rower #1) indicates that the training intensity distribution derived from TRAIN_≥*z1*_ did not considerably differ from TOTAL_≥*z1*_ in most weeks, while it did in weeks 20–26. In weeks 25–26, the lack of zone 3 and zone 4–5 during TRAIN_≥*z1*_ resulted in a polarization index of 0.00 a.U., but inclusion of OFF_≥*z*_ revealed a typical pyramidal TID for TOTAL_≥*z1*_. Similar, but mitigated results are displayed in Fig. 3b (rower ID 3). Again, TID calculated solely from TRAIN_≥*z1*_ is similar to TOTAL_≥*z1*_. However, substantial amounts of training in zone 4–5 during weeks 8, 20, and 24 accomplished during OFF_≥*z1*_ resulted in a different TOTAL_≥*z1*_ distribution.

### Performance data

**Figure 4:**
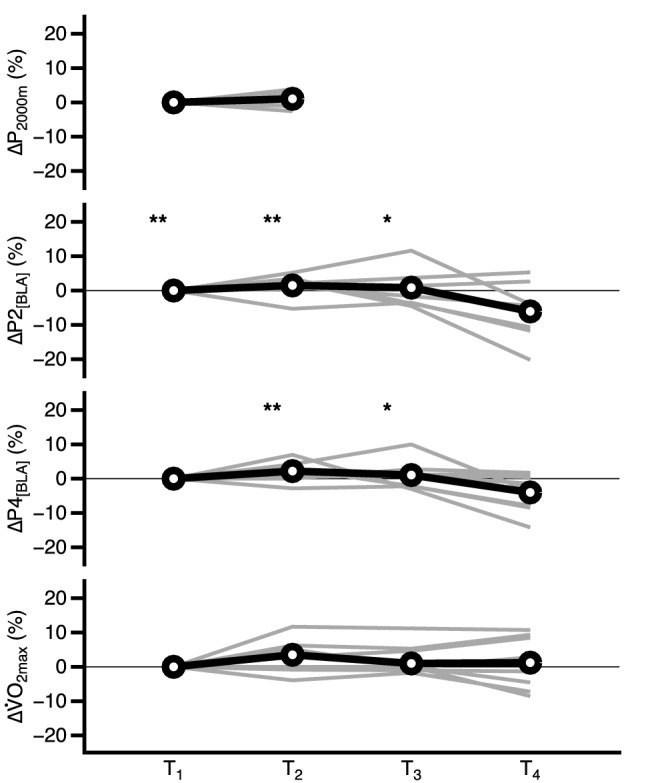
Individual (grey) and average (arithmetic mean in black and bold) percentage changes in performance and maximum oxygen consumption ($\dot{V}$O_2max_) of eight rowers within one season. T_1–4_ represent time points of measurement in November, March, May and around the individual rower’s main competition (July–September). *P*_*2000m*_ average mechanical power output during all-out 2000 m ergometer test, *P2*_*[BLA]*_* and P4*_*[BLA]*_ mechanical power output associated with blood lactate concentration of 2 or 4 mmol·L^−1^, respectively, $\dot{V}$*O*_*2max*_ maximum oxygen consumption. *Significant difference of absolute data to T_4_ with p < 0.05 (*) and **p < 0.01 (**) calculated with Tukey’s test in case of significant main effect.

The P2_[BLA]_ and P4_[BLA]_ of our rowers changed significantly over time (p = 0.027 and 0.031), while ${\dot{\text{V}}}$O_2max_ and P_2000m_ did not (p = 0.612 and 0.403), as shown in Fig. 4, which illustrates individual and average percentage changes in P2_[BLA]_, P4_[BLA]_ , ${\dot{\text{V}}}$O_2max_, and P_2000m_. The absolute mean values at T_1_, T_2_, T_3_, and T_4_ were 265 ± 21.1 W, 271 ± 21.4 W, 268 ± 21.3 W, and 248 ± 21.1 W for P2_[BLA]_, 307 ± 24.3, 316 ± 24.2, 311 ± 24.1, and 293 ± 24.0 for P4_[BLA]_, and 5.5 ± 0.45, 5.6 ± 0.45, 5.5 ± 0.46, 5.4 ± 0.45 for ${\dot{\text{V}}}$O_2max_. The average duration for the distance of 2000 m remained statistically unaltered (T_1_: 06:32.1 min ± 39.1 s to T_2_: 06:29.2 min ± 39.9 s; p = 0.404).

### Off-training and performance

Mixed model analysis revealed significant changes in P2_[BLA],_ P4_[BLA]_, and ${\dot{\text{V}}}$O_2max_ over time (all p ≤ 0.0001) but no effect of training volume per week, sessions per week, or TRIMPS per week on the changes in P2_[BLA],_ P4_[BLA]_, or ${\dot{\text{V}}}$O_2max_ for TOTAL, TRAIN, or OFF (p ≥ 0.072 ≤ 0.604, Supplementary Table S2). This result did not change when considering only the last 21 days before T_1_, T_2_, T_3_ and T_4_ (p ranging 0.091–0.662; Supplementary Table S3).

## Discussion

Here we aimed to evaluate the impact of activities performed during TRAIN_≥*z1*_ and OFF_≥*z1*_ on performance in eight elite athletes over a 30–45-week period. The main results were that OFF_≥*z1*_ contributed with more than 2 h·week^−1^, or 14–19%, to the total exercise volume per week. Further, 31% of all TRAIN sessions were performed with an intensity < zone 1. The intensity distribution of TRAIN did not significantly change when OFF_≥*z1*_ was included; however, for only 6 weeks and 4 individuals, a polarized training intensity distribution shifted to a non-polarized distribution or vice versa. Finally, our data did not reveal a different impact of TRAIN_≥*z1*_ or TOTAL_≥*z1*_ on changes in performance in this group of rowers.

The average training volume, including intensities below zone 1, amounted to 806 min·week^−1^, approximately 13% lower than the observed volume reported for U23-rowers in a previous study^1^, and also lower than the training volumes previously reported for elite rowers (900–1440 min·week^−1^)^6,23–26^. The difference between previous findings and our data is partly attributable to the fact that three of eight athletes in our study were not competing and training at the world-elite level—when excluding these data, the average volume increases to 905 min/week, which is in line with previous data. Differences from different training data collection approaches, such as time in zone vs. session goal, as demonstrated by Sylta et al.^19^ have little influence, but if training analysis is solely based on prescription instead of measurements (e.g.^6,27,28^) this may lead to an unclear reporting of either scheduled or actual training regarding volume and intensity.

An important finding of our study is that activities performed at ≥ 60% of maximum heart rate during waking hours outside of an actual training session (i.e., OFF_≥*z1*_) contributed with up to 14% of TRAIN and even 19% of TRAIN_≥z1_. Given that the difference from TRAIN_≥z1_ to TOTAL_≥z1_ is of a magnitude that can be considered relevant, because it discriminates the groups in some training studies^3,24^, this present result suggests including OFF_≥z1_ with TRAIN_≥z1_ when analyzing the influence of training (volume) on performance, as already proposed before^29^. However, our analysis did not reveal a significant effect of OFF on performance (Supplementary Tables S2, S3), which somewhat dampens our demand for the integration of OFF into training analysis. This result was surprising, because it suggests that additional low-intensity training during OFF_≥1_ may not provide a stimulus for physiological adaptions in this group, even though cellular metabolic effects of low intensity exercise also been reported also in highly trained athletes^30^. However, we advise caution in concluding from this first study of its kind that OFF activities are irrelevant. It is possible that relatively small longitudinal changes in performance (often within the smallest detectable change) contributed to the lack of significance, as did the relatively few performance measurements and the observational design of our study, which did not allow to clearly discriminate conditions of, for example, low vs. high volume during OFF. In addition, there are problems inherent with single day performance testing associated with confounding factors including hydration status, sleep quality, or motivation.

Notably, 31% of all TRAIN volume was performed with an intensity below zone 1. This is a remarkable result, because in endurance sports such as rowing, volume is generally considered a key component for success^6,31^, with the assumption that intensities are ≥ 60% HR_max_^5^. As a limitation, we acknowledge that the present TRAIN analysis also includes strength and other training components, in which heart rate is a poor surrogate for training load. However, Table 3 indicates that 18% of rowing and 35% of unspecific endurance training were performed with an intensity below zone 1. For both rowing and unspecific endurance training, low intensities (< 60 HR_max_) are potentially insufficient to impose any ergogenic disturbance in cardiovascular hemostasis^5^, especially in well-trained athletes. However, we are aware that such training can still be useful, because, for example, motor learning is often performed without relevant cardio-vascular or metabolic effort. Nevertheless, the relative high amount of training volume below zone 1 (i.e., < 60 HR_max_) during unspecific endurance training (mostly indoor and outdoor cycling) suggests that the rowers in our study might have not stimulated their cardiovascular system sufficiently during considerable proportions of their endurance training.

On the group level, the inclusion of OFF_≥*z1*_ to TRAIN_≥*z1*_ did not change TID of TOTAL_≥*z1*_ significantly, since most TRAIN_≥*z1*_ and OFF_≥*z1*_ activities were performed at low intensities in zone 1. On an individual level, however, our data indicate that OFF activities should be considered when assessing the TOTAL distribution. This notion is based on the observation of meaningful changes of the polarization index in some athletes by including or excluding OFF_≥*z1*_. Secondly, our data revealed that the maximum of additional high-intensity training (zone 3) during OFF_≥*z1*_ ranged 4–45 min·week^−1^ (95% CI) with an average of 21 min·week^−1^. In elite sports the individual case matters and an additional volume of 21 min·week^−1^ high-intensity or more must be considered in the daily decision-making process of training.

At this point, it is worth considering that the time in zone approach used in our study tends to underestimate the percentage of high-intensity exercise in comparison to the session goal approach^19^. However, the time in zone approach was appropriate for our purposes because it allows consistent combination of TRAIN and OFF, while the session goal method is not sensitive for OFF analysis. In any case, the plausibility of dominant low-intensity exercise during OFF in combination with a considerable amount of high or “vigorous” intensity and a trend towards polarized intensity distributions during OFF is supported by previous findings from our group that also reported “on or off” behavior in rowers outside of their regular training^9^.

Some general limitations also warrant acknowledgement. Though this is (to the best of our knowledge) the first study and dataset integrating OFF and TRAIN over several months as well as performance measures in such highly trained athletes, we were not able to capture *all* possible data during waking hours. In the present case, 23% of possible weeks were lost or rated as invalid because of synchronization problems, malfunctioning hardware updates, or battery and connectivity issues. Consequently, the lack of statistical differences between TRAIN and TOTAL training intensity distribution might be related to the missing data. Additionally, the final (small) sample size probably contributed to the lack of correlation between the effects of TRAIN and TOTAL on performance. In fact, some athletes dropped out of the study because they were annoyed by the frequent uploading and battery charging as well as several technical issues (e.g., data transmission, software updating) related to the smartwatch. Even those athletes who recorded data accurately lost considerable amounts of data due to unsuccessful data synchronization with the server. Nevertheless, we decided against including three of these athletes because it would have reduced the quality of the current data set. It is worth noting that wrist-worn sensor technology has improved significantly since 2018 (the year the study initiated), and technological issues will not be as much of a hindrance for future longitudinal studies.

The integration of training stimuli occurring during daily living—that is, outside of scheduled training—significantly altered the volume (14–19%) and training load (11%) in elite rowers, who exercised for approximately one-third of their weekly volume with intensities below 60% of their maximum heart rate, especially during unspecific endurance training. The training intensity distribution on a group level did not change when OFF activities were included. However, on an individual level, OFF activity may alter TOTAL intensity distribution. The present data set, which is to the best of our knowledge the first attempt to analyze OFF and TRAIN over several months with repeated performance measures in elite athletes, did not reveal a significant effect of such off-training activities on changes in performance surrogates including *P2*_*[BLA]*_*, P4*_*[BLA]*_, or ${\dot{\text{V}}}$O_2max_. However, based on our data we recommend adding information about OFF-training activity to TRAIN analyses, since this alters the data basis considerably and the lack of a significant association to performance changes in our study might be related to some of the limitations. Future research with more advanced monitoring technology is warranted to evaluate, if significant alterations in training descriptors allow for a more comprehensive understanding of the interaction of training stimuli and adaption.

## Supplementary Information

Supplementary Table S1.

Supplementary Table S2.

Supplementary Table S3.

## Data Availability

The datasets generated during and/or analyzed during the current study are available from the corresponding author on reasonable request.
