# Supplementary material for: The integration of training and off-training activities substantially alters training volume and load analysis in elite rowers
Source: Sci Rep. 2021 Aug 26;11:17218. doi: 10.1038/s41598-021-96569-0 (PMC8390693; doi:10.1038/s41598-021-96569-0)
Supplement: Supplementary file 1 — Supplementary Table S1. [file 41598_2021_96569_MOESM1_ESM.docx]

Table S1: Training intensity distribution and polarization index during TRAIN and OFF

| **Rower’s**  **ID** |  | **TRAIN** | |  | **OFF** | |
| --- | --- | --- | --- | --- | --- | --- |
|  |  | **TID%** | **Pol-Index a.U.** |  | **TID%** | **Pol-Index a.U.** |
| 1 |  | 90-6-4 | 1.78 |  | 95-4-2 | 1.68 |
| 2 |  | 87-8-6 | 1.81 |  | 95-2-4 | 2.28 |
| 3 |  | 96-3-2 | 1.81 |  | 99-0-0 | 2.00 |
| 4 |  | 94-2-4 | 2.27 |  | 95-2-3 | 2.15 |
| 5 |  | 61-24-15 | 1.58 |  | 84-5-12 | 2.30 |
| 6 |  | 91-6-4 | 1.78 |  | 94-5-1 | 1.27 |
| 7 |  | 95-2-3 | 2.15 |  | 94-2-4 | 2.27 |
| 8 |  | 79-15-5 | 1.42 |  | 87-2-11 | 2.68 |

*TRAIN = training or activity data recorded during scheduled training; OFF = training or activity data recorded outside of TRAIN; TID = training intensity distribution given as percentages spent in intensity zones 1 (60 ≤ 82% of maximum heart rate) – zone 2 (82 ≤ 88%) – zone 3 (88-100%); Pol-Index = polarization index according to Treff et al. (2019) ^21^, distinguishing not-polarized from polarized TID at threshold > 2.00 a.U.*
