# Supplementary material for: The integration of training and off-training activities substantially alters training volume and load analysis in elite rowers
Source: Sci Rep. 2021 Aug 26;11:17218. doi: 10.1038/s41598-021-96569-0 (PMC8390693; doi:10.1038/s41598-021-96569-0)
Supplement: Supplementary file 2 — Supplementary Table S2. [file 41598_2021_96569_MOESM2_ESM.docx]

Table S2: Level of significance regarding the effect of time and selected training variables on performance measures (power at 2 and 4 mmol/L blood lactate (P2_[BLa]_ and P4_[BLa]_) and maximal oxygen consumption (⩒O_2max_) in eight national and international elite rowers.

| **Variable** | **Effect** | **P2_[BLa]_ [W]** | **P4_[BLa]_ [W]** | **V̇O_2max_ [L/min]** |
| --- | --- | --- | --- | --- |
| Volume |  |  |  |  |
| TOTAL | time | 0.0001 | 0.0001 | 0.0001 |
|  | Volume | 0.5390 | 0.4518 | 0.9609 |
| TRAIN | time | 0.0001 | 0.0001 | 0.0001 |
|  | Volume | 0.5957 | 0.6221 | 0.2232 |
| OFF | time | 0.0001 | 0.0001 | 0.0001 |
|  | Volume | 0.7083 | 0.6044 | 0.6763 |
| Volume (> 60% maximal heart rate) | | |  |  |
| TOTAL≥z1 | time | 0.0001 | 0.0001 | 0.0001 |
|  | Volume ≥z1 | 0.9183 | 0.9184 | 0.2276 |
| TRAIN≥z1 | time | 0.0001 | 0.0001 | 0.0001 |
|  | Volume ≥z1 | 0.3456 | 0.4325 | 0.0891 |
| OFF≥z1 | time | 0.0001 | 0.0001 | 0.0001 |
|  | Volume ≥z1 | 0.1056 | 0.0829 | 0.4188 |
| Sessions |  |  |  |  |
| TOTAL≥z1 | time | 0.0001 | 0.0001 | 0.0001 |
|  | Sessions | 0.9055 | 0.7479 | 0.5472 |
| TRAIN≥z1 | time | 0.0001 | 0.0001 | 0.0001 |
|  | Sessions | 0.2176 | 0.2893 | 0.0723 |
| OFF≥z1 | time | 0.0001 | 0.0001 | 0.0001 |
|  | Sessions | 0.3557 | 0.2904 | 0.6803 |
| TRIMPS |  |  |  |  |
| TOTAL≥z1 | time | 0.0001 | 0.0001 | 0.0001 |
|  | TRIMPS | 0.7145 | 0.5540 | 0.3762 |
| TRAIN≥z1 | time | 0.0001 | 0.0001 | 0.0001 |
|  | TRIMPS | 0.8589 | 0.9919 | 0.2630 |
| OFF≥z1 | time | 0.0001 | 0.0001 | 0.0001 |
|  | TRIMPS | 0.1420 | 0.1141 | 0.5625 |

*TRAIN = training data recorded during scheduled training; OFF = training or activity data recorded outside of TRAIN; TOTAL = TRAIN + OFF; TRIMP = Training Impulse calculated as activity zone (1-5) x volume (minutes) adapted from Banister et al. (1975) ^20^. Effective volume = Training intensities > 60% of maximal heart rate. Data are based on mean values of all training data recorded between four performance measurements. See text for further details.*
