# Supplementary material for: The integration of training and off-training activities substantially alters training volume and load analysis in elite rowers
Source: Sci Rep. 2021 Aug 26;11:17218. doi: 10.1038/s41598-021-96569-0 (PMC8390693; doi:10.1038/s41598-021-96569-0)
Supplement: Supplementary file 3 — Supplementary Table S3. [file 41598_2021_96569_MOESM3_ESM.docx]

Table S3: Level of significance regarding the effect of time and selected training variables on power at 2 and 4 mmol/L blood lactate (P2_[BLa]_ and P4_[BLa]_) and maximal oxygen consumption (V̇O_2_max) in eight national and international elite rowers.

| **Variable** | **Effect** | **P2_[BLa]_ [W]** | **P4_[BLa]_ [W]** | **V̇O_2max_ [L/min]** |
| --- | --- | --- | --- | --- |
| **Volume** |  |  |  |  |
| TOTAL | time | 0.000 | 0.004 | 0.000 |
|  | Volume | 0.292 | 0.337 | 0.203 |
| TRAIN | time | 0.000 | 0.001 | 0.000 |
|  | Volume | 0.798 | 0.940 | 0.395 |
| OFF | time | 0.000 | 0.004 | 0.000 |
|  | Volume | 0.279 | 0.380 | 0.132 |
| **Volume** (> 60% maximal hear rate) | | |  |  |
|  |  |  |  |  |
| TOTAL*_≥z1_* | time | 0.000 | 0.001 | 0.000 |
|  | Volume ≥z1 | 0.442 | 0.571 | 0.248 |
| TRAIN*_≥z1_* | time | 0.000 | 0.001 | 0.000 |
|  | Volume ≥z1 | 0.712 | 0.555 | 0.891 |
| OFF*_≥z1_* | time | 0.000 | 0.001 | 0.000 |
|  | Volume ≥z1 | 0.091 | 0.097 | 0.105 |
| **Sessions** |  |  |  |  |
| TOTAL*_≥z1_* | time | 0.000 | 0.001 | 0.000 |
|  | Sessions | 0.865 | 0.928 | 0.662 |
| TRAIN*_≥z1_* | time | 0.000 | 0.001 | 0.000 |
|  | Sessions | 0.236 | 0.157 | 0.469 |
| OFF*_≥z1_* | time | 0.000 | 0.001 | 0.000 |
|  | Sessions | 0.474 | 0.459 | 0.437 |
| **TRIMPS** |  |  |  |  |
| TOTAL*_≥z1_* | time | 0.000 | 0.002 | 0.000 |
|  | TRIMPS | 0.346 | 0.433 | 0.182 |
| TRAIN*_≥z1_* | time | 0.000 | 0.001 | 0.000 |
|  | TRIMPS | 0.948 | 0.953 | 0.642 |
| OFF*_≥z1_* | time | 0.000 | 0.001 | 0.000 |
|  | TRIMPS | 0.142 | 0.155 | 0.159 |

TRAIN = training or activity data recorded during scheduled training; OFF = training or activity data recorded outside of TRAIN; TOTAL = TRAIN + OFF; TRIMP = Training Impulse calculated as activity zone (1-5) x volume (minutes) adapted from *Banister et al. (1975) ^20^.* Effective volume = Training intensities > 60% of maximal heart rate.

Data are based on 21-day mean values of training data preceding the four performance measurements. See methods for further details.
